# When to Scaffold Motivational Self-Regulation Strategies for High School Students' Science Text Comprehension

**DOI:** 10.3389/fpsyg.2021.658027

**Published:** 2021-05-14

**Authors:** Tova Michalsky

**Affiliations:** Bar-Ilan University, Ramat Gan, Israel

**Keywords:** metamotivation scaffolding, motivational regulation strategies, science literacy, science knowledge, microbiology texts

## Abstract

Noting the important role of motivation in science students' reading comprehension, this 14-weeks quasi-experiment investigated the optimal timing for implementation of metamotivational scaffolding for self-regulation of scientific text comprehension. The “IMPROVE” metamotivational self-regulatory model (Introducing new concepts, Metamotivation questioning, Practicing, Reviewing and reducing difficulties, Obtaining mastery, Verification, and Enrichment) was embedded at three different phases of secondary students' engagement with scientific texts and exercises (before, during, or after) to examine effects of timing on groups' science literacy and motivational regulation. Israeli 10th graders (*N* = 202) in eight science classrooms received the same scientific texts and reading comprehension exercises in four groups. Three treatment groups received metamotivational scaffolding before (*n* = 52), during (*n* = 50), or after text engagement (*n* = 54). The control group (*n* = 46) received standard instructional methods with no metamotivational scaffolding. Pretests and posttests assessed science literacy, domain-specific microbiology knowledge, and metamotivation regulation. Intergroup differences were non-significant at pretest but significant at posttest. The “before” group significantly outperformed all other groups. The “after” group significantly outperformed the “during” group, and the control group scored lowest. Outcomes suggested delivery of metamotivational scaffolding as a potentially important means for promoting students' science literacy and effortful perseverance with challenging science tasks, especially at the reflection-before-action stage for looking ahead and also at the reflection-on-action stage for looking back. More theoretical and practical implications of this preliminary study were discussed to meet the growing challenges in science teaching schoolwork.

## Introduction

Reading comprehension of scientific texts is a well-recognized, powerful vehicle for engaging students' minds and helping them construct scientific inquiry habits, reach a deep conceptual understanding, and attain science achievements (Graesser et al., 2002; Krajcik and Sutherland, 2010; Pearson et al., 2010; Yore and Tippett, 2014; van Rijk et al., 2017; Sason et al., 2020). However, researchers have asserted that the mere provision of reading opportunities and strategies is often insufficient to effectively develop science literacy without explicit scaffolding to support readers' self-regulated learning (Lai et al., 2014; Murphy et al., 2017). The Programme for International Student Assessment (PISA) defines science literacy as “the ability to engage with science-related issues, and with the ideas of science as a reflective citizen” (Organisation for Economic Co-operation Development, 2015a, p. 50).

Motivation plays an important role in science students' reading by determining the extent to which students engage with science texts and persevere in applying effort, without aborting, until successfully completing the texts' accompanying reading comprehension tasks (see review by Morgan and Fuchs, 2007). Thus, when facing a reading task, students must not only attain knowledge about reading comprehension strategies such as locating ideas in text and processing and integrating information—namely, *cognitive* self-regulation skills—but also must attain knowledge about how and when to apply these different cognitive strategies—namely, *metacognitive* self-regulation skills (e.g., Schreiber, 2005; Roebers, 2017; Jian, 2018; Pamungkas et al., 2018; Farhana et al., 2020). Students must also acquire explicit strategies for self-regulating their own *motivation*—deciding how to approach the knowledge acquisition process and how much effort to invest—to cope with what may be a cognitively, emotionally, and temporally demanding task (e.g., McClelland et al., 2007; Guthrie and Coddington, 2009; Kelley and Decker, 2009; Logan et al., 2011; Skibbe et al., 2011; Liew et al., 2019; Li et al., 2020).

Most prior research on learners' self-regulation in the reading context has focused on supporting the cognitive and metacognitive aspects of self-regulated learning (also see Ahmadi et al., 2013; Rastegar et al., 2017; see reviews in Ali and Razali, 2019 and Deliany and Cahyono, 2020). In contrast, the motivational aspect has been under-investigated (McNamara, 2017; Egloff, 2019; Egloff and Souvignier, 2020). Embedment of self-questions into learning material to guide students' autonomous or collaborative self-regulation of their own learning processes (e.g., Kramarski and Mevarech, 1997 “IMPROVE” method) has been shown effective for enhancing science and math learners' cognitive and metacognitive self-regulation as well as for promoting their academic achievements (Michalsky, 2013, 2020; Michalsky and Schechter, 2018). However, most prior research on self-questioning supports has been conducted in the context of cognitive and metacognitive self-regulation components rather than the motivational self-regulation component.

At the focus of the current study, previous research has not yet sufficiently explored when best to specifically embed motivational scaffolding to maximize science learners' active engagement in, and comprehension of, reading tasks. Although scaffolding to support self-regulated learning processes may be implemented at different chronological phases of learning—at the forethought, performance, or retrospective self-reflection phases (Zimmerman, 1990)—little attention has been given in the empirical literature to the relative effectiveness of the before-task *vs*. during-task *vs*. after-task timeframe for delivering motivational regulation support for scientific text reading. The current study aims to narrow this gap by expanding the literature on the benefit of scaffolding for the motivational aspect of self-regulated learning in a science reading context and, in particular, on the design of its optimal conditions.

## Metamotivation Processes for Scientific Text Reading

Motivational self-regulation, otherwise termed “metamotivation,” refers to the conscious processing of monitoring and controlling one's own motivation to increase effort and persistence when completing a task or achieving a particular learning goal (McNamara, 2017; Schwinger and Otterpohl, 2017). Specifically, motivational regulation includes two main component processes: monitoring and control (Corno, 1993; Boekaerts, 1995; Kuhl, 2000; Wolters, 2003, 2011; Pintrich, 2004; Sansone and Thoman, 2005, 2006; Schwinger and Stiensmeier-Pelster, 2012). Metamotivational “monitoring” refers to self-awareness or self-evaluation of one's motivation, whereas metamotivational “control” refers to self-management and self-regulation of one's motivation and efforts (Zeidner and Stoeger, 2019), operating as reciprocal processes that form a feedback loop (Miele and Scholer, 2018).

*Metamotivational monitoring* refers to students' evaluation of the quantity and the intrinsic or extrinsic quality of their own motivation to achieve a goal or complete a task. In the context of science text comprehension, students must be motivated to invest effort in trying to understand the written material and in monitoring their comprehension to observe lapses in understanding (Oakhill and Cain, 2012; Dutke et al., 2016; De Smedt et al., 2020). Such monitoring includes recognizing what motivated one to read about a science topic in the first place, what decreases one's motivation, how one's current motivation can be informed by motivation in prior similar tasks, and what one still needs to monitor so as to remediate discrepancies in one's motivation to read. According to Meniado (2016), the development of science reading comprehension skills is significantly better for learners who monitor their reading motivation.

*Metamotivational control* refers to choosing and actively performing strategies that strengthen or shift one's motivation. Such strategies include the use of self-talk to regulate efforts and actions, environmental control efforts to establish external conditions that are more conducive to learning effectively, and “self-consequating” behaviors, where students promise themselves a reward or reinforcement after achieving their academic goal (Schwinger and Otterpohl, 2017). Students who manage their motivation while reading—by taking a more active interest in the topic, finding a personal connection to the material, trying to reduce outside distractions, and mobilizing attention to decipher difficult ideas presented in the text—have reported experiencing a more successful learning process, gaining a sense of satisfaction and enjoyment from learning, and understanding the study topics better (Kamil et al., 2008; Salinger, 2010; Schwinger and Otterpohl, 2017).

In the context of comprehending written scientific content, metamotivational monitoring can encourage students to think about (i.e., to self-evaluate) what motivates them to comprehend a text. Having identified their reasons, readers can then take actions to self-manage (i.e., control) their engagement with the task, with the aim of increasing their motivation and, in turn, their reading comprehension (Bråten et al., 2013). For example, Azhari (2020) has recently reported that monitoring of reading motivation is a driving factor that encourages students to manage and meet their expressed goals. The experiments of Nguyen et al. (2019) have also demonstrated that, to manage motivational states effectively, students must at minimum possess self-awareness or self-evaluation about which states would be more/less advantageous for a particular task and how to produce them.

Thus, to promote students' reading comprehension and achievements, researchers have strongly underscored the importance of explicitly training students to both monitor and regulate their own motivational processes (Reynolds, 2017; Schwinger and Otterpohl, 2017). However, prior research has not yet sufficiently investigated when best to embed explicit metamotivational scaffolding to enhance learners' active, effective engagement with science reading comprehension tasks and to promote their science literacy.

## Timing of Metamotivational Self-Questioning Support

Schon (1996) distinguishes between *in-action* and *on-action* reflective self-questioning. *Reflection-in-action* describes interaction with a “live” problem as it unfolds during task performance—also termed by Raelin (2001) as “contemporaneous reflection,” occurring at the moment. *Reflection-on-action* describes activation of reflection processes after task performance, which enables learners to construct and evaluate explicit theories of action for solving future scientific problems—termed by Raelin (2001) as “retrospective reflection” for looking back at the experience. According to Seibert (1999), students tend to deal with live problems spontaneously, using their tacit knowledge, even when problems elicit uncertainty or surprise; hence, at the in-action (during-task) phase, reflection processes are generally not activated. In contrast, on-action (after-task) reflection processes are activated whenever a problem contains ambiguity or conflict because learners must consciously confront their tacit theories of action to evaluate their problem solution.

While Schon's work is highly regarded, it does not refer to the activation of before-action reflection processes (Hackett, 2001). *Reflection-before-action* has been described as a “pre-reflection” stage (Dewey, 1997) or as “anticipatory reflection,” often occurring at the planning stage (Raelin, 2001). In pre-task reflection, teachers may provide students with self-directed questions that give external structure to the self-regulation process in the form of a general work routine.

Researchers have begun to construct innovative instructional methods for science students based on metamotivational reflection as supported by self-directed questioning (Puteh and Ibrahim, 2010; Salinger, 2010; Schwinger and Stiensmeier-Pelster, 2012; Bråten et al., 2013; Michalsky, 2013; Frankel, 2016). However, empirical studies to date have not yet simultaneously compared the three possible metamotivational scaffolding timeframes corresponding with the forethought, performance, and retrospection phases of self-regulated learning of Zimmerman (1990).

Research to date examining before-task scaffolding for self-regulated learning, delivered only at the reflection-before-action phase (per Dewey, 1997; Raelin, 2001), has not sufficiently investigated science reading or scaffolding that specifically focused on metamotivation. In the math and literature learning contexts, pre-task scaffolding given to high-school students to support their cognitive and metacognitive (but not motivational) self-regulated learning (i.e., before math problem solving or literature reading comprehension) has been found to promote learners' motivation to perform the task as well as their academic-domain achievements (Mevarech and Kramarski, 2017; Reynolds, 2017). These findings for before-task cognitive and metacognitive scaffolding suggest the possible benefits of pre-action self-questioning of a metamotivational nature. Scholer and Miele (2016) have recommended using metamotivation processes before the learning action to enhance learners' fuller engagement in tasks and higher self-efficacy for successfully activating cognitive processes, compared with conditions where metamotivation is used during and or after cognitive processes.

Little research has examined the efficacy of retrospective metamotivational self-questioning in the form of instructional scaffolding presented to learners only after completing their task (reflection-on-action per Schon, 1996). In one study, Scholer and Miele (2016) have shown that scaffolding for metamotivation reflection delivered after completing a science learning task improves university students' achievements, compared with students who do not receive such scaffolding.

With regard to during-task scaffolding, delivered only at the reflection-in-action phase (per Schon, 1996), comparative research has shown that high school students exposed to metamotivational self-questioning instructional methods during their reading of microbiology texts significantly outperform their peers who do not receive metamotivational self-questioning support, as assessed on measures of general science literacy and domain-specific microbiology knowledge (du Boulay, 2011; Mahdavi and Tensfeldt, 2013; Michalsky, 2013).

In one of the rare studies to date comparing the three timeframes for self-regulatory scaffolding of reading comprehension, Michalsky (2013) has presented IMPROVE self-questions to elementary school students before, during, and after reading science texts (albeit addressing metacognitive regulation, not motivational regulation). Findings have indicated that these younger children perform best when they receive the metacognitive self-questioning after their text reading, as assessed by measures of general and domain-specific science achievements and metacognitive awareness. Prior research has also shown improvements in all three components of self-regulation [cognition, metacognition, and motivation as assessed using the measure of Pintrich (1999)] when secondary school students adapt their trained reading methods to a specific text *via* the use of a preplanned external procedure that helps structure students' processes of cognitive and metacognitive (but not motivational) self-regulation as implemented before, during, and after the reading task (Souvignier and Mokhlesgerami, 2006).

## Current Study Objectives

In line with the call of Lajoie (2005) to examine not only what and how to scaffold for reading comprehension but also when to scaffold and when to fade scaffolding, the current study's preliminary exploration aims to narrow the gaps mentioned earlier in the literature. The present quasi-experiment compared the effectiveness of the three timeframes for introducing metamotivational self-questioning support—before, during, or immediately after reading scientific texts—as compared with a control group receiving no metamotivational support at all. To comprehensively assess the impact of the four different intervention conditions, gains were measured in general science literacy, domain-specific knowledge, and metamotivational skills following the intervention.

This study focused on 10th graders because, during adolescence, students' engagement in and motivation for reading decrease (Wigfield et al., 2016). Specifically, with regard to the field of science literacy, students often report a strong dislike of reading, which may be attributed to age-related decreases in self-efficacy beliefs about their adequacy of skills and knowledge for comprehending increasingly complex secondary school science texts (Guthrie and Coddington, 2009; Cunningham and Zibulsky, 2014).

Based on prior literature indicating that supporting students' motivation for reading enhances scientific text comprehension (e.g., Scholer and Miele, 2016; Reynolds, 2017), all three metamotivational groups were expected to outperform the control group on all three dependent variables. Due to the paucity mentioned earlier of comparative research regarding the timing of metamotivational scaffolding for secondary students' scientific text comprehension (see, for example, rare studies on motivation and cognition aspects by Souvignier and Mokhlesgerami, 2006; Logan et al., 2011), no explicit assumption was formulated about the effects of the “before” vs. “during” vs. “after” metamotivational scaffolding approaches embedded at three different phases of the scientific text reading.

## Method

### Participants

Participants were 202 10th graders (102 boys, 100 girls; mean age: 15.5 years, *SD* = 0.63) who attended eight heterogeneous classes belonging to different districts. The classes were randomly selected from 21 Israeli high schools whose science teachers participated in a long-term 4-months in-service training program concerning the 10th-grade *Invitation to Scientific Inquiry* science curriculum. The following parameters were similar across all high schools: size (two to three classes per grade level for grades 7–12), middle-class socioeconomic status as defined by the Israel Ministry of Education (Central Bureau of Statistics, 2006), and students' pretest science achievement levels.

The eight teachers who underwent the current training and delivered the intervention to their 10th-grade science classes (five females, three males; mean age: 35 years, *SD* = 0.82) held a science teaching certification, an academic degree in science, and more than 8 years of experience in science teaching. Two teachers each were randomly assigned to the four intervention conditions: those receiving metamotivational self-questioning support before text reading (“BEF,” *n* = 52), during text reading (“DUR,” *n* = 50), or after text reading (“AFT,” *n* = 54), and those who did not receive any self-questioning or metamotivational support (“control,” *n* = 46). Statistical analyses conducted at pretest on demographic variables for teachers (sex, age, and years of teaching experience) and students (sex and age) and on all study variables for students yielded no statistically significant intergroup differences.

## Science Study Unit

All students in all eight classrooms studied the “The World of Microorganisms” study unit for 3.5 months during 10th grade as part of a series of science study units entitled *Invitation to Scientific Inquiry* (National Research Council, 2015). All eight classrooms used the same textbook and read the exact same scientific texts comprising the inquiry-based “Microbiology” unit for three lessons per week over 14 weeks. The experiment was delivered in six of the eight classrooms, in only one of their three weekly microbiology lessons. Once weekly, the six teachers in the “BEF,” “DUR,” and “AFT” groups (two teachers per group) implemented their assigned metamotivational self-questioning scaffolding method for their 10th graders' reading comprehension of scientific texts, whereas the two teachers in the control condition used traditional instruction for all three weekly lessons.

For the first 2 weeks of the 14-weeks period, the two classrooms comprising the control group introduced standard instructional methods for scientific text comprehension, with no metamotivational scaffolding. In the remaining six of the eight classrooms, the teachers in the three experimental groups dedicated their first two weekly lessons to introducing their assigned IMPROVE self-questioning model (BEF, DUR, or AFT) that students would utilize before, during, or after reading scientific texts, respectively. During these two introductory training lessons, the instructors in the three experimental groups provided demonstrations and modeling of their respective metamotivational scaffolding conditions to initially train students in the utilization of these scaffolds for attempts to monitor and manage their motivation and efforts.

For all four learning conditions, each of the remaining 12 lessons contained three parts: outline, practice, and summary phases (see Appendix A). In the practice phase that comprised most of each lesson (~30 min), all students practiced comprehension of the lesson's scientific texts and, based on their reading, worked to solve related scientific exercises. Each student in all four groups received a personal copy of the printed worksheet presenting that lesson's microbiology text and accompanying exercises. The scientific texts were related to the microbiology phenomena that all students were learning in their other two weekly classes. For the three treatment conditions only, the worksheets additionally presented the metamotivational scaffolds, differing between the BEF, DUR, and AFT groups only in the timing of their embedment.

As shown in Table 1, for all four groups, each round of engagement with a science reading comprehension task comprised two phases: (a) individual reading and individual exercise performance, followed by (b) small-group discussion and joint reflection on the individual students' exercise solutions. Only in the BEF/DUR/AFT conditions (but not in the control condition) did students receive and respond to metamotivational training scaffolds during these two engagement phases.

**Table 1:** Four groups' training conditions (Modeled in lessons 1 and 2 and practiced in lessons 3 and 14).

	**Lesson phase**	**Treatment groups**			**Control group**
		**BEF: Before reading** **(*n* = 52)**	**DUR: During reading** **(*n* = 50)**	**AFT: After reading** **(*n* = 54)**	
Training procedure	A. Individual work	1. Self-questions 2. Text reading & exercises	Text reading & exercises, alternating with self-questions	1. Text reading & exercises 2. Self-questions	Text reading & exercises
	B. Small-group discussion	1. Self-questions 2. Exercises	Exercises, alternating with self-questions	1. Exercises 2. Self-questions	Exercises
General focus of responses to self-questions during training		Expectations about anticipated text/topic, not associated with a specific case	Specific difficulties or successes arising from the experience	The global experience and less so to the details	Not applicable

“*Self-questions,” for the three treatment groups only, included: (1) four IMPROVE metamotivation self-questions on Comprehension, Connection, Strategy, and Reflection, embedded into printed worksheets according to conditions' timing, to scaffold the microbiology text reading and its accompanying comprehension exercises; and (2) a printed card cueing eight-strategy metamotivation management repertoire to support the Strategy self-question*.

## Three Experimental Metamotivational Treatment Conditions

Students in the three experimental conditions (BEF, DUR, and AFT) were all exposed to the same series of four self-addressed metamotivation questions, based on the IMPROVE method of Kramarski and Mevarech (1997) as updated by Michalsky (2013). These four self-questioning scaffolds for each microbiology reading comprehension task pertained to Comprehension (task knowledge), Connection (inter-task knowledge), Strategies (strategy knowledge), and Reflection (self-knowledge). The three experimental training groups' metamotivational scaffolds differed from one another only in (a) the timing of their embedment in the worksheets (e.g., see Appendix B for the DUR group) and (b) their use of appropriate tense: future tense for the BEF group, present tense for the DUR group, and past and future tense for the AFT group (see left column of Table 2).

**Table 2:** IMPROVE metamotivational self-questions, with sample excerpts from individual phase of student training lessons, by treatment group.

**IMPROVE metamotivational self-questions**	**Excerpts from individual metamotivational practicing by experimental group**		
	**BEF: Before reading** **(*n*** **= 52)**	**DUR: During reading** **(*n*** **= 50)**	**AFT: After reading** **(*n*** **= 54)**
**Comprehension (task knowledge)** State and explain: What will motivate/is motivating/motivated your performance of the scientific text reading and comprehension exercises? Why? Please explain your reasoning. When running into difficulties, what will you do?	• Knowing that the exercise will help me understand much better what we are learning in class with the teacher. • I've long been interested in science topics. • I've always wondered how HIV infects people and why it's so dangerous.	• Trying to answer the questions about the virus life cycle successfully. • The truth is, I don't know why I have to read this difficult text on sterilized rats. Low motivation.	• It really helped me to understand more thoroughly what we learned in class. • It was really interesting. • I had no motivation at all.
**Connection (inter-task knowledge)** What will be/are/were the similarities and differences between your motivation and efforts in the reading and comprehension exercises at hand compared to those you have solved in the past?	• Because I read about bacteria before, I think this text will be easier for me. • I have never heard about this subject, so it looks interesting.	• There are a lot of concepts and ideas that are not familiar to me, so it is lowering my motivation. Why isn't it easier? • This time I made a flowchart of what I am reading, so it's giving me a good feeling and helping me gain a lot of motivation.	• This time I was less motivated because the topic wasn't that interesting for me. • I arrived with less motivation but was very surprised that I was able to understand. In the previous task, the topic was very promising, but I was bored. This time the opposite happened. In the end, it was interesting.
**Strategies (strategy knowledge)** What strategies from the repertoire you learned in class or which other strategies do you plan to use/are you using/did you use in performing the reading and comprehension exercises? Why?(Use your printed card cueing the eight-strategy metamotivation management repertoire)	• I will remind myself that in the end, we have a common task that needs to be solved together. • I will tell myself not to give up on putting in more effort because I will then be able to answer the questions at the end and succeed in the sciences.	• I read the whole part on the mechanism of bacterial resistance to antibiotics, sentence by sentence because it is important for me to succeed in answering the questions at the end. • I just skip the hard vocabulary words about the biological lifecycle of the virus at first, so I do not have to stop reading. I will go back to them at the end.	• I constantly read out loud to myself to stay focused, so I could succeed later when everyone answered the questions in the team. And I also talked to myself, so I could get a high grade. • I saw that everyone was reading, so I realized that this is a text that can be managed alone and that I would be able to succeed and not be left behind.
**Reflection (self-knowledge)** Do you feel good about your motivation and efforts for the reading and comprehension exercises that you are going to perform/are performing/performed? Explain.	• I am not sure… But I am beginning to convince myself that it is important for my success in school. • I am going in with a positive attitude to reading this text.	• I lose a lot of my motivation because I run into difficulties and can't read fluently. So how can I get motivated in this kind of situation? • I am satisfied with my level of motivation to persevere in the task. I can raise my motivation if I think about my future success in the science field.	• I had a lot of motivation to read because I finally understood what probiotics are, and I have to remember that there are a lot of topics that if you do not start to delve into them, then you would not really understand and would not enjoy them. • I lost motivation in the middle because I remembered we had a science test tomorrow. Just a shame I didn't concentrate on the task. I should have told myself that the more I concentrate, the more I will succeed on the test tomorrow.

During the two introductory training lessons (Lessons 1 and 2), the instructors in the three experimental groups demonstrated to students how to utilize the four IMPROVE self-questioning scaffolds throughout their individual and small-group phases of engagement with the study unit's assigned science texts and to accompany reading comprehension exercises, according to each condition's timing for embedment (see Table 1).

During reading task practice for the experimental conditions in Lessons 3–14, the individual engagement phase was accompanied by an individual responding to the four metamotivational self-questioning scaffolds (see Table 2 for excerpts from students' utilization of these training scaffolds in the individual phase). Then, the small-group engagement phase in the three groups was accompanied by joint discussion and reflection on their metamotivational responses.

To be noted, the Comprehension, Connection, and Reflection self-questions scaffolded students' motivational monitoring or awareness, whereas the Strategy question uniquely scaffolded students' attempts to control and manage their motivation and efforts. Thus, to scaffold the training procedure for the Strategy question in each lesson, each student in all three treatment conditions also received a personal copy of a user-friendly printed card cueing them about the repertoire of eight possible strategies for managing their own motivation (e.g., how to apply self-talk in their science reading task; see left column of Table 3). The instructor had modeled and exemplified these eight motivational management strategies in Lessons 1 and 2, based on Schwinger and Otterpohl (2017), according to each condition's timing for the Strategy self-question. Each treatment condition's worksheets and cards were included in the teachers' guidebook.

**Table 3:** Repertoire of eight motivational management strategies.

	**Strategy**^***a***^	**Motivational regulation strategies questionnaire (Schwinger et al.**, **2009****)**		
		**No. of items**	**Cronbach α**	**Sample items**
1	Enhancement of personal significance	3	0.75	I strive to relate the scientific text to my own experiences
2	Mastery self-talk	4	0.83	I persuade myself to keep on reading to find out how much I can read scientific text successfully
3	Enhancement of situational interest	5	0.86	I make reading scientific text more pleasant for me by trying to arrange it playfully
4	Performance-approach self-talk	5	0.80	I call my attention to the fact of how important it is to obtain good grades
5	Performance-avoidance self-talk	3	0.87	I imagine that my classmates make fun of my poor performance
6	Environmental control	4	0.79	Before beginning with work, I strive to eliminate all possible distractions
7	Self-consequating	3	0.77	I make a deal with myself, saying that I will do something pleasant after I finish work
8	Proximal goal setting	3	0.87	I approach work step-by-step in order to get the feeling that I am progressing well
	Total	30	0.84	

a *Strategies listed in left column were presented on a printed card to each student in three treatment groups throughout training procedure, cueing eight-strategy metamotivation management repertoire to scaffold IMPROVE Strategy self-question*.

## Teacher Training

To prevent treatment diffusion and compensatory rivalry, each pair of teachers (in the BEF, DUR, AFT, and control conditions) participated in a separate 2-days (6-h) in-service training program on the instruction of scientific text comprehension. The training instructor (the author) holds expertise in science text reading comprehension and the different metamotivational support conditions.

The first day of training was the same for all four conditions (BEF, DUR, AFT, and control), emphasizing the importance of strengthening students' science literacy and discussing the possible difficulties students encounter in comprehending scientific texts. The second day differed according to the assigned condition. The two teachers in the control group received the standard national 10th-grade approaches for reading science texts relevant to the study unit, and the instructor demonstrated teaching methods for enhancing scientific text comprehension in the classroom. Each of the three metamotivational conditions (two teachers each) received an introduction to the rationale and techniques of their assigned IMPROVE scaffolding method (BEF, DUR, or AFT). For these six teachers, the instructor accentuated the importance of metamotivation for encouraging scientific text reading, demonstrated the assigned timing and procedure for the IMPROVE self-questions' implementation in the classroom, and also modeled the use of the eight motivational management strategies based on Schwinger and Otterpohl (2017).

Throughout both days of training for all eight teachers, the instructor strongly emphasized the benefit of encouraging students to initiate discourse with their small-group team members through instructions such as: “Discuss your scientific ideas and reasoning with your team” or “Explain your answers to your peers.”

## Fidelity

To ensure teachers' adherence to the scaffolding methods, all eight classrooms were observed by the author every other week across the 12-weeks experiment (8 classes × 6 observations = 48 observations in total). For the three treatment conditions (BEF, DUR, and AFT), observations were conducted in the one weekly biology lesson (out of three) when the teachers applied their assigned metamotivational scaffolding. For the control group, the observed lessons were selected randomly. After every observation, the instructor gave feedback to each observed teacher, answered teachers' questions, and offered recommendations for improvement if necessary. Overall, the teachers adhered well to the training they had received, both (a) regarding the microbiology unit's correlation to the standard national science curriculum and pedagogic inquiry strategies and (b) regarding their assigned scaffolding approach (or none) for reading scientific texts.

## Assessment Measures

Three measurements were each completed by students at the pretest and posttest intervals.

### Domain-Specific Microbiology Test

This 22-item domain-specific test was designed by the National Science Committee of the Israel Ministry of Education (2015) to examine students' knowledge of “The World of Microorganisms” science curriculum. The test included 10 multiple-choice items such as “Which of these statements is not true about HIV?” giving the following four choices [the correct response is “c”]: (a) It has a long incubation time, causing it to be able to remain in the host for a long time before discovery; (b) It is a retrovirus; (c) It infects all human cells; (d) There is a high rate of mutation in the HIV virus. Each of the 10 multiple-choice items was scored as either 0 (incorrect) or 4 (correct), with the total score for these items ranging from 0 to 40.

The scoring for each of the test's 12 open-ended questions (e.g., “Write two viruses' characteristics”) ranged from 0 (incorrect) to 5 (full answer), with the total score for all open-ended questions ranging from 0 to 60. Two trained judges with expertise in science knowledge coded students' responses. Inter-judge reliability, calculated for the same 35% of the responses coded by both judges, yielded reliability coefficients ranging from *r* = 0.88 to 0.97 for all levels. Total microbiology scores were 0–100. The correlation between the pretest and posttest scores was *r* = 0.81.

### General Test of Science Literacy

This 15-item test was designed for the purpose of the current study based on PISA 2015 science literacy tests (Organisation for Economic Co-operation Development, 2017), tapping into students' “literacy” in the five major components of scientific experiments (see Table 4). Two trained judges with expertise in science knowledge coded students' responses. Inter-judge reliability, calculated for the same 40% of the responses coded by both judges, yielded reliability coefficients ranging from *r* = 0.81 to 0.93 for all levels. Total general literacy scores were 0–100. The correlation between the pretest and posttest scores was *r* = 0.85.

**Table 4:** Sample items for five components on 15-item general test of science literacy.

**Literacy component**	**Sample from two closed multiple-choice items:** **Scored either 0 (incorrect) or 7 (correct)**	**One open-ended item:Scored either 0 (incorrect) or 6 (full answer)**	**Cronbach α**
Describing phenomena	Bacteria do not develop in honey. Why? (a) Bacteria do not like sweets. (b) Viscosity of the honey does not allow colonies to be created. (c) Honey does not contain nutrients for bacteria. (d) (correct answer) Bacteria dry out and die.	After the experiments conducted by the students in pickling cucumbers and making yogurt, Adina stopped eating olives, pickled cucumbers, and yogurt. She claimed that these products contain bacteria, and bacteria can cause disease. Introduce a counterargument that might persuade Adina to eat these products again.	0.82
Formulating hypotheses	What will happen to a small number of bacteria transferred to a closed vessel containing food and optimal temperature conditions? (a) bacteria immediately multiply at a rapid rate thanks to the abundance of food. (b) In a closed vessel, bacteria will not be able to multiply at all. (c) (correct answer) Number of bacteria will increase as long as there is enough food and oxygen. (d) Number of bacteria will increase more and more despite the pH change in the vessel.	Healthy humans' digestive system has a very large number of bacteria. Our body's immunology systems do not work against them. Why? Suggest an experiment for testing the resistance of those bacteria. Address the following issues: Formulate a hypothesis for testing the question and explain the basis for your hypothesis.	0.87
Identifying dependent variables	Researchers were asked to estimate the number of bacteria in a fixed volume of a given solution. Each researcher chose a different method to count the bacteria. In which of the following counting methods will the smallest number of bacteria be found? (a) Counting under a microscope. (b) Counting using a device that checks the degree of turbidity. (c) In all methods, the same number of bacteria will be counted. (d) (correct answer) Culturing the solution and counting colonies of bacteria.	What is the dependent variable in the suggested experiment?	0.86
Identifying independent variables	A grain of soil contains a diverse population of bacteria. If you want to increase the percentage of bacteria performing photosynthesis out of all the bacteria in the soil grain, it is advisable to transfer the soil grain to: (a) A lighted food substrate, which contains organic compounds. (b) (correct answer) Illuminated food substrate, which does not contain organic compounds. (c) A food substrate in the dark, which contains organic compounds. (d) A food substrate in the dark, which does not contain organic compounds.	What is the independent variable in the suggested experiment?	0.83
Reporting the results and drawing conclusions	Here are some facts about bacteria that occur in the process of acidification. Mark the facts that explain the cucumber pickling and yogurt making processes: (a) These bacteria are tiny creatures that have one cell and lack a nucleus. (b) These bacteria feed on organic substances found in their environment. (c) (correct answer) These bacteria carry out the process of anaerobic respiration (agitation). The decomposition products are acid and carbon dioxide. (d) These bacteria multiply in the process of division, which explains the cucumber pickling and yogurt-making processes.	Which results support your hypothesis? What conclusions can you draw from those results?	0.81
Total score range: 0–100	For closed items: 0–70	For open items: 0–30	0.84

### Motivational Regulation Strategies in Reading Science Texts

Students' self-reported use of metamotivational management efforts was assessed using the 30-item Motivational Regulation Strategies Questionnaire of Schwinger et al. (2009), adapted to the specific context of reading scientific literature. Sample items and reliabilities for the eight different motivational regulation strategies are presented in Table 3.

## Procedure

The research reported in this study involving human participants was approved by the Research Ethics Board at Bar-Ilan University in accordance with ethical standards comparable to the 1964 Helsinki declaration. The eight participating teachers in the current study were randomly selected from 21 teachers who volunteered for further training and research on scientific text comprehension, following their participation in the *Invitation to Scientific Inquiry* in-service training program held in central Israel. The eight teachers were then randomly assigned to one of the four intervention conditions (two teachers each to the BEF, DUR, AFT, and control conditions). The purpose of the study and the existence of the other intervention conditions were masked; teachers were only informed by the training instructor that they were participating in an experiment on new pedagogical approaches to enhance scientific text comprehension.

All students were administered the three pretests (on science literacy, domain-specific microbiology knowledge, and motivational regulation strategies for reading science texts) during their biology lessons within the first 3 weeks of the school year, immediately before beginning the 3-months “The Microorganisms' World” science learning unit. At the end of the 3-months unit, all students completed the three measures again in their biology classrooms (posttests).

## Data Analyses

One-way within-subject analyses of variance (ANOVA) with repeated measures were conducted, with treatment (four groups) as the independent variable and with posttest performance measures (for the three tests separately) as the dependent variables. Analyses of the total scores were followed by analyses of component subscales. *Post hoc* comparisons were conducted as needed in the form of pairwise contrasts. In addition, correlations were calculated among the three dependent variables at the end of the study (Time 2) for each of the four research groups.

## Results

### Domain-Specific Science Knowledge Test on Microbiology

Table 5 presents the means, standard deviations, and adjusted means for students' total scores on the Test of Science Knowledge by time and treatment. As seen on the table, at pretest, no significant differences emerged between the treatment groups with regard to microbiology knowledge, *F*_(1,201)_ = 13.56, η^2^ = 0.19, *p* > 0.18. This validated the four groups' equivalent baseline scores for microbiology knowledge. The one-way repeated measures ANOVA revealed a significant main effect for Time, *MS*_*e*_ = 5.3, *F*_(1,201)_ = 11.3, η^2^ = 0.36, *p* < 0.001, and a significant Time × Treatment interaction, *F*_(1,201)_ = 31.2, η^2^ =0.15, *p* < 0.001. However, at the posttest interval, significant intergroup differences did emerge. *Post hoc* analyses of the adjusted mean scores based on pairwise comparison *t*-tests indicated that the BEF group (*M* = 78.31) significantly outperformed all other groups; the AFT group (*M* = 70.32) significantly outperformed the DUR group (*M* = 74.11); and the control group (*M* = 65.17) attained the significantly lowest microbiology knowledge scores (all *p* < 0.05).

**Table 5:** Students' means, standard deviations, and adjusted mean scores on the test of domain-specific science knowledge, by time (pre/post) and treatment.

**Microbiology knowledge**	**Group**							
	**Treatment: Metamotivational scaffolding**						**Control: No scaffolding** **(*****n*** = **46)**	
	**BEF: Before reading** **(*****n*** = **52)**		**DUR: During reading** **(*****n*** = **50)**		**AFT: After reading** **(*****n*** = **54)**			
	**Pre**	**Post**	**Pre**	**Post**	**Pre**	**Post**	**Pre**	**Post**
*M*	46.63	78.66	49.53	70.14	47.31	74.82	47.72	66.05
(Adj. *M*)		78.31		70.32		74.11		65.17
*SD*	10.12	14.18	11.28	13.42	11.45	13.26	12.34	14.25
Scores ranged from 0 to 100.								

### General Science Literacy

**Figure 1:**
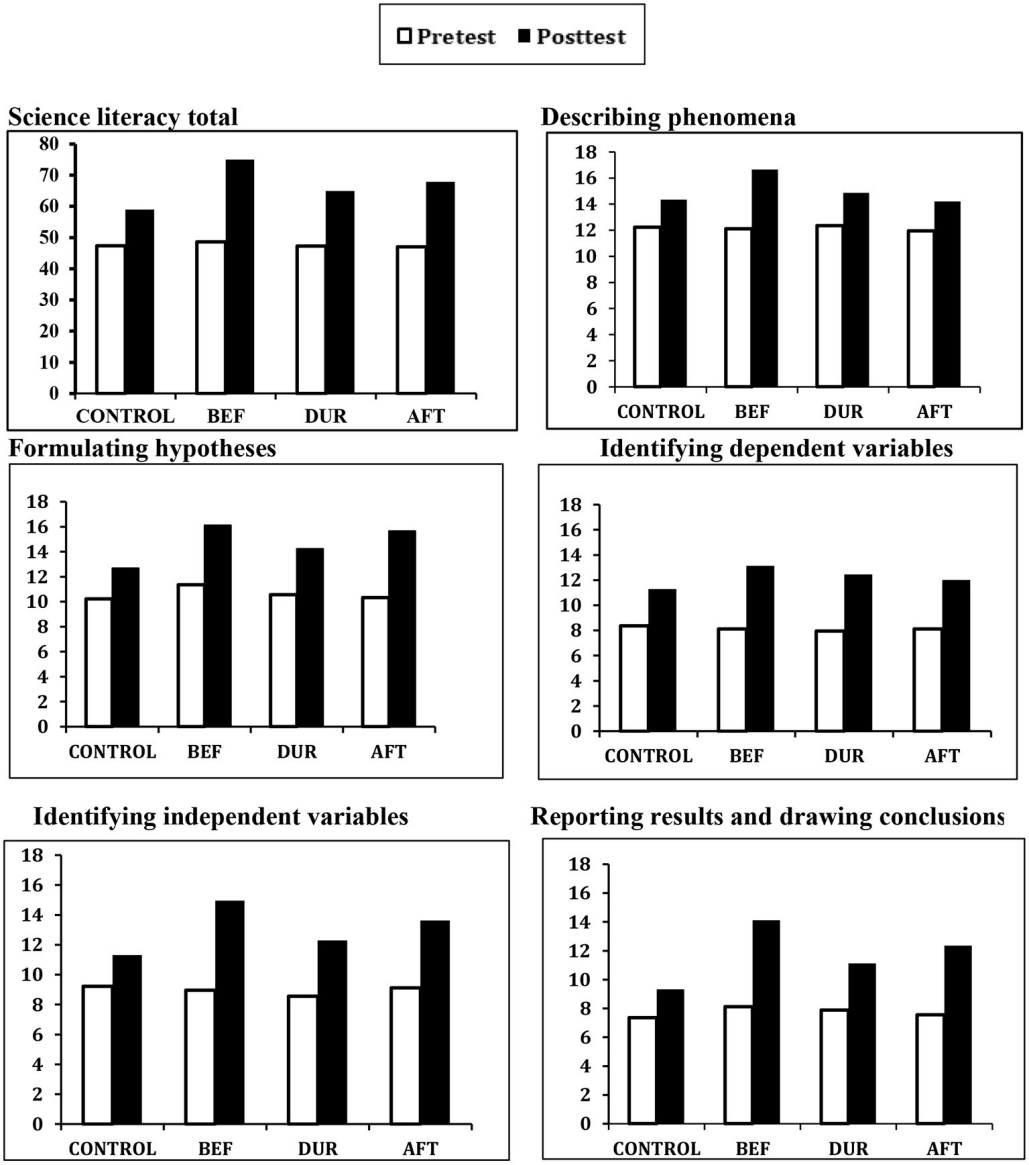
Mean scores on science literacy total and components, by time and treatment. BEF = metamotivation intervention before text reading; DUR = metamotivation intervention during text reading; AFT = metamotivation intervention after text reading; Control = no metamotivation intervention.

Table 6 presents the means, standard deviations, and adjusted means for students' total scores and subscale scores on the Test of Science Literacy by time and treatment. At pretest, no significant differences emerged between the treatment groups regarding the total score or any of the five components of general science literacy, *F*_(1,201)_ = 18.32, η^2^ = 0.13, *p* > 0.22. This validated the four groups' equivalent baseline scores for general science literacy. As presented on the table, the one-way repeated measures ANOVAs revealed a significant main effect for Time and a significant Time × Treatment interaction for the total score, *MS*_*e*_ = 25.16, *p* < 0.001, and for all five of the literacy components, *MS*_*e*_ = 28.36, *p* < 0.001. As illustrated in Figure 1, *post hoc* analyses of the adjusted mean scores based on the pairwise comparison *t*-test indicated that on the total score, the BEF group (*M* = 74.98) significantly outperformed all other groups; the AFT research group (*M* = 62.96) significantly outperformed the DUR group (*M* = 67.84); and the control group (*M* = 58.98) attained the significantly lowest mean literacy scores (all *p* < 0.05). As seen in the figure, the same pattern of findings emerged for all five science literacy components.

**Table 6:** Means, standard deviations, and cohen's d effect sizes^a^ on general test of science literacy, by time and treatment, with significant effects.

**Literacy component**	**Group**								**Significant effects (*****p*** **<** **0.001)**			
	**Treatment: Metamotivational scaffolding**								**Time**		**Time** **×** **Treatment interaction**	
	**BEF: Before reading** **(*****n*** = **52)**		**DUR: During reading** **(*****n*** = **50)**		**AFT: After reading** **(*****n*** = **54)**		**CON: Controls—No scaffolding (*****n*** **=** **46)**					
	**Pre**	**Post**	**Pre**	**Post**	**Pre**	**Post**	**Pre**	**Post**	***F*_**(1,201)**_**	***η***^***2***^	***F*_**(1,201)**_**	***η***^***2***^
**DESCRIBING PHENOMENA**												
*M*	12.11	16.65	11.92	14.86	12.35	14.21	12.23	14.35	97.12	0.41	24.17	0.47
*SD*	3.93	3.51	3.94	4.12	4.03	4.62	4.11	5.23				
*d*	1.26		0.49		0.62		0.46					
**FORMULATING HYPOTHESES**												
*M*	11.36	16.11	10.56	14.23	11.32	15.65	10.23	12.69	67.45	0.41	27.37	0.51
*SD*	3.13	3.21	3.41	3.32	3.61	3.42	2.91	3.12				
*d*	1.53		1.11		1.23		0.82					
**IDENTIFYING DEPENDENT VARIABLES**												
*M*	8.12	13.14	7.96	12.05	8.11	12.41	8.36	10.50	74.69	0.53	39.36	0.44
*SD*	3.42	3.51	3.93	3.91	3.52	3.44	3.71	3.81				
*d*	1.47		1.05		1.15		0.56					
**IDENTIFYING INDEPENDENT VARIABLES**												
*M*	8.96	14.96	8.55	12.30	9.12	13.62	9.23	11.32	145.35	0.57	64.36	0.55
*SD*	2.62	2.43	3.11	2.83	2.80	2.65	3.34	3.23				
*d*	*2.4*1		1.25		1.61		0.65					
**REPORTING RESULTS AND DRAWING CONCLUSIONS**												
*M*	8.12	14.16	7.88	11.12	7.55	12.35	7.36	9.32	102.00	0.66	32.69	0.53
*SD*	3.82	3.74	3.73	3.75	3.12	3.14	3.13	3.23				
*d*	1.90		0.90		1.54		0.62					
**TOTAL FOR SCIENCE LITERACY**												
*M*	48.67	74.98	47.34	62.96	47.08	67.84	47.41	58.98	112.32	0.42	35.63	0.38
*SD*	8.12	8.31	7.31	7.17	8.53	8.22	8.43	8.51				
*d*	3.22		2.03		2.51		1.43					

*Scores ranged from 0 to 20 for each of the five components and from 0 to 100 for the total. Significant differences emerged for all five components (p < 0.001): BEF > AFT, DUR, CON; AFT > DUR, CON; DUR > CON*.

a *Cohen's d effect size was calculated as the ratio between the posttest minus the pretest value and the average standard deviation of the pretest*.

### Motivational Regulation Strategies for Science Text Reading

Table 7 presents the means, standard deviations, and adjusted means for students' total scores and eight strategy scores on the Motivational Regulation Strategies Test, by time and treatment. At pretest, no significant differences emerged between the treatment groups regarding the total score or any of the eight strategies, *F*_(1,201)_ = 18.63, η^2^ = 0.23, *p* > 0.24. This validated the four groups' equivalent baseline scores on motivational regulation strategies. As presented in the table, the one-way repeated measures ANOVAs revealed a significant main effect for Time and a significant Time × Treatment interaction for the total score, *MS*_*e*_ = 36.12, *p* < 0.001, and for all eight motivational management strategies, *MS*_*e*_ = 23.25, *p* < 0.001. *Post hoc* analyses of the adjusted mean scores based on the pairwise comparison *t*-test indicated that on the total score, the BEF group (*M* = 4.12) significantly outperformed all other groups; the AFT group (*M* = 3.24) significantly outperformed the DUR group (*M* = 3.77); and the control group (*M* = 2.91) attained the significantly lowest mean scores in motivational regulation (all *p* < 0.001). As seen in Table 7, the same pattern of findings emerged for all eight strategies.

**Table 7:** Means, standard deviations, and cohen's d effect sizes^a^ of motivational regulation strategies, by time and treatment, with significant effects.

**Self-regulation strategy**	**Treatment group: Metamotivational scaffolding**						**Controls—**		**Significant effects (*****p*** ***<*** **0.001)**			
	**BEF: Before reading** **(*****n*** = **52)**		**DUR: During reading** **(*****n*** = **50)**		**AFT: After reading** **(*****n*** = **54)**		**CON: No scaffolding** **(*****n*** = **46)**		**Time**		**Time** **×** **Treatment interaction**	
	**Pre**	**Post**	**Pre**	**Post**	**Pre**	**Post**	**Pre**	**Post**	***F*_**(1,201)**_**	***η***^***2***^	***F*_**(1,201)**_**	***η***^***2***^
**ENHANCEMENT OF SITUATIONAL INTEREST**												
*M*	3.12	4.64	2.92	3.44	3.04	3.92	2.91	3.52	74.37	0.55	32.14	0.43
*SD*	1.34	1.51	1.32	1.42	1.33	1.32	1.21	1.22				
*d*	1.15		0.38		0.69		0.46					
**ENHANCEMENT OF PERSONAL SIGNIFICANCE**												
*M*	2.61	3.92	2.71	3.03	2.54	3.51	2.60	3.11	91.36	0.52	43.12	0.29
*SD*	1.32	1.52	1.24	1.41	1.62	1.53	1.41	1.32				
*d*	1.00		0.25		0.62		0.35					
**MASTERY SELF-TALK**												
*M*	3.52	4.73	3.24	4.01	3.42	4.55	3.32	3.81	84.39	0.33	47.39	0.56
*SD*	1.41	1.52	1.23	1.32	1.34	1.44	1.36	1.51				
*d*	0.85		0.66		0.84		0.38					
**PERFORMANCE-APPROACH SELF-TALK**												
*M*	2.51	3.62	2.22	3.04	2.44	3.45	2.33	2.82	125.13	0.41	52.39	0.52
*SD*	1.45	1.94	1.23	1.94	1.32	1.83	1.32	1.74				
*d*	0.78		0.66		0.76		0.38					
**PERFORMANCE-AVOIDANCE SELF-TALK**												
*M*	2.43	3.82	2.67	3.38	2.54	3.45	2.31	2.82	98.12	0.55	47.63	0.42
*SD*	1.21	1.43	1.51	1.31	1.37	1.34	1.36	1.44				
*d*	1.16		0.90		0.69		0.38					
**SELF-CONSEQUATING**												
*M*	2.42	4.13	2.24	3.03	2.21	3.44	2.33	2.82	144.19	0.49	44.35	0.52
*SD*	1.42	1.51	1.23	1.31	1.34	1.42	1.33	1.52				
*d*	1.21		0.94		1.21		0.38					
**ENVIRONMENTAL CONTROL**												
*M*	2.23	3.92	2.44	3.43	2.41	3.62	2.34	2.85	122.32	0.47	51.36	0.52
*SD*	1.32	1.41	1.23	1.22	1.31	1.41	1.46	1.55				
*d*	1.30		0.83		0.92		0.35					
*M*	2.42	3.91	2.33	2.84	2.22	3.12	2.43	2.73	97.36	0.42	35.63	0.38
*SD*	1.12	1.31	1.22	1.24	1.50	1.26	1.44	1.57				
*d*	1.22		0.43		0.62		0.24					
**TOTAL FOR MOTIVATIONAL REGULATION**												
*M*	2.63	4.12	2.60	3.24	2.05	3.77	2.43	2.91	125.77	0.57	42.36	0.51
*SD*	1.12	1.31	1.24	1.25	1.51	1.48	1.41	1.50				
*d*	1.25		0.54		0.92		0.32					

*Scores ranged from 1 to 5 for each of the eight strategies and for the total. Significant differences emerged for all eight strategies (p < 0.001): BEF > AFT,DUR,CON; AFT > DUR,CON; DUR > CON*.

a *Cohen's d effect size was calculated as the ratio between the posttest minus the pretest value and the average standard deviation of the pretest*.

### Correlations Among Dependent Variables at Time 2

Table 8 presents the results of the correlation analysis conducted among science literacy, domain-specific microbiology knowledge, and metamotivation regulation strategies for each of the four research groups at the end of the study. Significantly higher correlations (using Fisher's transformation of *r* to *Z*) were found in the BEF group than in the other three groups. The AFT group revealed significantly higher correlations than the DUR group. The control group showed the lowest correlations among dependent variables, which were all non-significant.

**Table 8:** Correlations (Fisher's transformation of r to Z) among dependent variables in the four research groups at time 2.

	**General science literacy**				**Domain-specific microbiology achievements**			
	**BEF**	**DUR**	**AFT**	**Control**	**BEF**	**DUR**	**AFT**	**Control**
Motivational regulation	0.47^*^	0.27	0.36^*^	0.16	0.55^*^	0.32^*^	0.42^*^	0.24
General science literacy	—	—	—	—	0.57^**^	0.28^*^	0.39^*^	0.24

*BEF: Before reading (n = 52); DUR: During reading (n = 50); AFT: After reading (n = 54); Control: No scaffolding (n = 46)*.

* *p < 0.05*.

** *p < 0.01*.

## Discussion

Findings from the current quasi-experiment clearly highlighted the advantage of metamotivational scaffolding's embedment in 10th-graders' scientific text reading over the effectiveness of standard instructional methods that do not include any such scaffolding. Namely, as expected, all three student groups who received support for motivational self-regulation were found to outperform the control group on all studied variables: not only on their ability to regulate their own motivation to read about science but also on their general and domain-specific science achievements. With regard to the main focus of this study—identifying the optimal phase for embedding metamotivational scaffolding—high school students who received such scaffolding before the reading task significantly outperformed the other two groups who received metamotivational scaffolding either during or after reading, regarding all of the outcomes assessed in the present study. More extensive consideration is given next to these findings.

### Benefit of Metamotivational Scaffolding Over Standard Instruction

The advantage found for metamotivational scaffolding (in the BEF, DUR, and AFT groups) over standard instructional methods (in the control group) coincides with prior studies showing that explicit scaffolding is a necessity when training students to self-regulate their motivation while reading scientific texts (Souvignier and Mokhlesgerami, 2006; Michalsky, 2013; Hsu et al., 2016; McNamara, 2017). This outcome also substantiates the claim that mere exposure to scientific texts is insufficient on its own (Ozuru et al., 2009). As Hartman (2001, p. 56) has argued [emphasis appeared in the original]:

Teachers should not be satisfied with putting students in situations which require them to use any strategy they want students to use. **Practice isn't enough**. It is also important to provide explicit instruction in **when, why and how** to use the strategy; students need to understand the rationale and effective procedures for the strategy so that they can recognize appropriate contexts for its use, so that they have criteria for evaluating their strategy, and so they can self-regulate its use.

The three treatment groups' higher gains in motivational self-regulation than the control group may be attributed to the reflective processes inherent in answering the self-addressed metamotivation questions. Namely, contemplating the Comprehension, Connection, and Reflection self-questions may have promoted students' self-awareness of their own motivation, whereas contemplating the Strategy self-question and cued repertoire of strategies may have promoted their self-management of that motivation. These metamotivational monitoring and control processes (Veenman et al., 2006) may, in turn, facilitate students' science achievements.

### Benefits of Metamotivational Scaffolding Given Before Science Text Engagement

The BEF group of 10th graders exposed to metamotivational scaffolding before they began reading each text and its accompanying comprehension exercises significantly outperformed the other two metamotivation groups (DUR and AFT). This advantage for the BEF group occurred, although all three treatment groups had received the same training and scaffolds (for self-regulatory motivational reflection *via* the four IMPROVE self-questions and for metamotivational management *via* the eight-strategy repertoire) at some point in their engagement with the same science texts and exercises.

This current finding on high school students differs from similar prior research outcomes focused on the metacognitive rather than motivational component of self-regulation among younger students (Michalsky, 2013). Further research is needed to determine if the different outcomes (highest effectiveness of pre-reading scaffolding for secondary students in the current study vs. highest effectiveness of post-reading scaffolding for elementary students in Michalsky, 2013) may possibly be attributable to factors related to students' age and/or to the metacognitive vs. metamotivation type of self-questioning scaffolding. For example, perhaps the adolescents' age-related cognitive abstraction capability or short-term working memory (Souza and Oberauer, 2016) may have enabled the high schoolers to maintain the IMPROVE self-questions in mind while engaging in their reading tasks, whereas the cognitive load may have been too heavy for those younger children who received before-task scaffolding in Michalsky (2013).

It may be speculated that answering the motivation-oriented self-questions before approaching the reading task may have served to focus the current 10th-grade BEF group's attention onto their motivational state across the entire ensuing reading context. Such initial mapping of their metamotivational monitoring may thereby have helped them to identify later when they were, or were not, experiencing an optimal motivational state for learning (Brown et al., 2016). Such better self-awareness, beginning in the starting phase of the learning task, in turn, may have fostered their ability to search for effective strategies and actions among their learned repertoire of metamotivational strategies (i.e., metamotivational control), to induce that optimal state in themselves all along with the upcoming reading task and comprehension exercises (Pintrich, 2002; Wolters, 2003; Miele and Scholer, 2018).

In addition, according to the chronological model of self-regulated learning phases of Zimmerman (2000), the “starting” forethought phase involves planning strategies such as task analysis or goal setting and is mainly influenced by learners' self-efficacy (belief in their competence, Bandura, 1977) regarding the learning task. Students with high self-efficacy have been shown to work diligently to master difficult scientific reading tasks, using their cognitive strategies productively (Zimmerman and Schunk, 2013). Perhaps, the early timing of the metamotivational self-questions in the BEF group enhances 10th-graders' optimism and confidence in their ability to cope with potential difficulties that may arise during engagement with the upcoming challenging science task. It may be that building up high self-efficacy leads, in turn, to better control over their own ensuing motivations (Butler et al., 2017). Considering self-efficacy beliefs' documented links to strategy use, self-regulation, and intrinsic motivation in the reading context (Pintrich and De Groot, 1990; Zimmerman and Schunk, 2003), researchers would do well to include self-efficacy measures in future metamotivational methodologies.

Not only the BEF group but also the AFT group outperformed the DUR group on all three dependent variables—motivational regulation strategies, general science literacy, and domain-specific science achievements. This resembled a prior finding for metacognitive scaffolding given during science text reading to elementary school students (Michalsky, 2013). Perhaps the lowest outcomes for the scaffolding provided during reading comprehension processes were attributable to learners' heavy cognitive load in this instruction condition. Bunch and Earl Lloyd (2006) have argued that cognitive load theory and cognitive load management are fundamental in reading comprehension because science texts provide large and complex amounts of information. Cognitive load theory posited that effective scaffolding facilitates learning by “directing cognitive resources toward activities that are relevant to learning rather than toward preliminaries to learning” (Chandler and Sweller, 1991, p. 294). Chandler and Sweller have noted that unnecessarily forcing learners to work with disparate sources of mutually referring information leads to ineffective scaffolding and to an increase in their cognitive load during reading. Therefore, scaffolding learners to utilize specific instructional materials before commencing their learning, as given in the current BEF group, may allow learners the freedom to employ any of the scaffolds at any time as they deem necessary to promote their understanding and solving of the problem.

The groups' patterns of correlations after the experiment—among their motivational regulation strategies, general science literacy, and domain-specific science achievements using the Fisher transformation—appear to corroborate the advantage found for the BEF reading group (showing the highest correlations) over the other three groups, followed by the AFT, DUR, and control groups, respectively. Although these correlational data do not permit assumptions about causality, stronger relationships may attest to more effective reciprocal influences between students' motivational self-regulation for science reading and their science literacy and educational achievement outcomes. Greater motivational regulation may lead to greater effort and persistence, which result in better instructional performance and *vice versa* (Bandura, 1977; Guthrie and Coddington, 2009). In this sense, the scaffolding of students' motivation at the before phase of reading science texts appears to have the greatest value for leveraging the important links between metamotivation, reading comprehension, and science achievements.

### Study Limitations, Implications

As a preliminary exploration of the timing of metamotivational scaffolding, the current study requires future validation. This study's utilization of only one self-questioning method (IMPROVE) suggests that future researchers would do well to expand investigation on timing to various additional kinds of metamotivational scaffolding methods such as prompts, teacher tutoring, and so on. Likewise, the stronger benefit of metamotivational scaffolding at the before phase of microbiology text reading should be examined regarding the diverse scientific content matter and non-science domains. Future researchers may also wish to scrutinize the role played by text difficulty, domain familiarity, and prior knowledge on how students utilize metamotivational scaffolding provided at different learning phases. To further explore scaffolding methods, a fine-grain inquiry may also help identify the relative effectiveness of the different metamotivational management strategies for enhancing science students' outcomes.

Furthermore, considering that the current outcomes contradict those found for elementary school children, the same methodology should be used simultaneously with students across age groups to elucidate developmental trajectories while also examining sex differences. The present study did not show any sex differences on any of the study variables, but prior research has indicated that girls tend to outperform boys on reading comprehension, whereas boys have a distinctive advantage over girls with regard to scientific interest and literacy (e.g., Organisation for Economic Co-operation Development, 2015b). Finally, qualitative methods such as think-aloud processes rather than quantitative self-reports may help clarify metamotivation experiences, skills, and strategies at different phases of text reading.

## Conclusions

Although no explicit assumptions could be formulated about the comparative effectiveness of the three timeframes due to the paucity of research in this area, the current preliminary study's outcomes highlight the potential impact of the current metamotivation instructional framework. Especially when delivered at the reflection-before-action stage for looking ahead and also at the reflection-on-action stage for looking back, metamotivational scaffolding may offer important means to promote science students' capacities and to meet the growing challenges in science teaching schoolwork.

## Data Availability Statement

The original contributions presented in the study are included in the article/Supplementary Material, further inquiries can be directed to the corresponding author/s.

## Ethics Statement

The studies involving human participants were reviewed and approved by Bar Ilan University Ramat-Gan 5290002. Written informed consent to participate in this study was provided by the participants' legal guardian/next of kin.

## Author Contributions

All authors listed have made a substantial, direct and intellectual contribution to the work, and approved it for publication.

## Conflict of Interest

The author declares that the research was conducted in the absence of any commercial or financial relationships that could be construed as a potential conflict of interest.
