# Supplementary material for: When to Scaffold Motivational Self-Regulation Strategies for High School Students' Science Text Comprehension
Source: Front Psychol. 2021 May 14;12:658027. doi: 10.3389/fpsyg.2021.658027 (PMC8160371; doi:10.3389/fpsyg.2021.658027)
Supplement: Supplementary file 1 [file Data_Sheet_1.pdf]

Appendix A

Intervention Lessons 3–14: Outline, Practice, and Summary Phases

Lessons' Outline Phase

First, in the initial ~5 minutes of each lesson, the teacher outlined the lesson's subject and contents to the students in the classroom, as related to various aspects of microbiology and general science literacy.

Lessons' Practice Phase

For the three metamotivation-training conditions, IMPROVE self-questioning scaffolding was embedded in the science reading task (differing in timing for the BEF, DUR, and AFT conditions), but no scaffolding was embedded in the text reading for the control condition.

All students' practice occurred first individually and then collaboratively with peers. Each student used a written manual to proceed through the individual and group practice steps autonomously, given oral supports from the teacher for time allotments. Students selected their own small groups at the start of the year and were asked to switch to a new group approximately every 6 weeks.

Specifically, to initially acquaint students with the learning activity, students were instructed to individually read the lesson's first scientific text and to solve its accompanying reading comprehension exercises in writing. For the three experimental conditions (not the control group), students were also asked to answer the metamotivational self-questions in writing, at some point in their individual work. Self-questions were embedded in the three treatment groups' worksheets and supported by a printed cue card of metamotivational management strategies supplied in their manual. Next, in small groups of 3-4 participants, students reflected together on their prior thinking processes from the individual stage, regarding their individual problem-solving of the reading comprehension exercises (in all four groups), and additionally regarding their metamotivational answers (in the experimental conditions only). In the small groups, participants were encouraged to collaborate with peers in reflective discourse while interpreting the text, predicting difficulties, and examining solutions for the accompanying scientific tasks. Groups submitted products representing members' agreement on exercises' solutions.

After completing the individual and group work regarding the lesson's first text and its accompanying tasks (and the metamotivational self-questions in the BEF/DUR/AFT conditions), this process repeated itself for additional science texts.

Lessons' Summary Phase

After completing the practicing core of each lesson, the teacher presented a summary of the lesson in the classroom. In these last ~10 minutes of the lesson, the teacher addressed any difficulties that may have arisen.
