# Supplementary material for: When to Scaffold Motivational Self-Regulation Strategies for High School Students' Science Text Comprehension
Source: Front Psychol. 2021 May 14;12:658027. doi: 10.3389/fpsyg.2021.658027 (PMC8160371; doi:10.3389/fpsyg.2021.658027)
Supplement: Supplementary file 2 [file Data_Sheet_2.pdf]

# Appendix B

*Sample Microbiology Worksheet for Students in the "DUR" (During) Condition, Alternating Text and Exercises with Four IMPROVE Metamotivational Self-Questions*

***Part A***

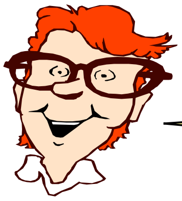

**What is your motivation for performing the reading and comprehension exercises?**
**(IMPROVE comprehension prompts)**

**Text**:
Many microorganisms exist in the intestines of rats and other healthy creatures. Pasteur (1822-1895) stated that the presence of some microbes is essential for the normal life of the organism. In recent years, researchers have found a way to breed rats that are free of microbes by using strict safeguards during the prenatal period. Researchers raised young rats in sterilized rooms, in which they breathed sterilized air, and were fed sterilized food and water. Researchers sought to determine if the amoebae, which causes entamoeba histolitica, also causes this disease in microbe-free rats.

**Task**:
A.1 Formulate a hypothesis for testing the question and explain the basis for your hypothesis.
A.2 Plan an experiment that confirms your hypothesis.
A.3 Describe the results that confirm your hypothesis. Explain.
A.4 In your opinion, is it worthwhile to use microbe-free rats in the experiments that you have just planned?

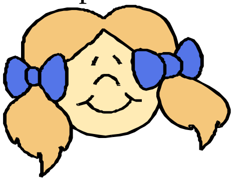

**What are the similarities/differences between your motivation in the reading and comprehension exercises at hand in comparison to those you have solved in the past?**
**(IMPROVE connection prompts)**

***Part B***

**Text**:
Researchers conducted an experiment and used 30 microbe-free rats and 30 regular rats of the same species and the same age. In each of these two groups, there were an identical number of males and females. All the rats were contaminated with amoebae and observed for several days. After this period of observation, researchers found the symptoms of the disease in 95% of the regular rats; researchers did not find any symptoms of the disease in the microbe-free rats.

**Task**:
B.1 Identify procedural components of the experiment.
B.2 Do the results of the experiment support or refute the hypothesis that you phrased in Question A.1? Discuss.
B.3 Are the results of the experiment compatible with Pasteur's statement? Discuss.
B.4 What can you conclude from the results of the experiment? Discuss.

***Part C***

**Text I:**
Microbe-free rats are more vulnerable to other infectious diseases than regular rats (unlike the case of contamination with the amoebae).

**Task**:
C.1 Suggest an explanation for the regular rats' resistance to infectious diseases. This resistance is greater than the resistance in the microbe-free rats.

C.2 According to the above information (in Text I), test your conclusion in Question B.4. If this information supports your conclusion, give reasons. If the information refutes your conclusion, suggest an explanation for this contradiction.

C.3 Assume what interrelationships exist between the microbes and the amoebae in the regular rats' intestines. Explain the microbe-free rats' resistance to the amoebae.

C.4 Would you recommend administering antibiotics to the regular rats that were infected with amoebae? Discuss.

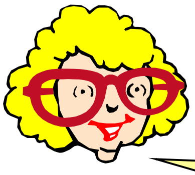

**What motivational strategies from the repertoire that you learned are you using now during the reading comprehension exercises? Why?**
**[Use your printed card cueing the 8-strategy metamotivation management repertoire]**
**(IMPROVE strategy prompts)**

**Text II:**

When regular rats are fed a vitamin deficient diet (lacking biotin and vitamin K), they survive for quite a long time, whereas within a few days the microbe-free rats show typical symptoms of vitamin deficiency, and die within two weeks.

**Task**:

C.5 What conclusions do you draw from this information about the importance of microbes in rats' intestines? Discuss.

C.6 Suggest an experiment for testing the influence of microbes in rats' intestines. Address the following issues:

a. What is your hypothesis? What is the independent variable in the suggested experiment?

b. What is the dependent variable in the suggested experiment?

c. Which results support your hypothesis?

C.7 Following the information that you learned in Text II, review the discussion you had in Question C.3. What is your opinion about this discussion in light of this new information?

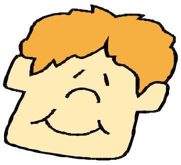

**Do you feel good about your motivation while reading and comprehending? Can you motivate yourself in another way? How? Explain.**
**(IMPROVE reflection prompts)**
